# Shape matters: effects of silver nanospheres and wires on human alveolar epithelial cells

**DOI:** 10.1186/1743-8977-8-36

**Published:** 2011-12-30

**Authors:** Linda C Stoehr, Edgar Gonzalez, Andreas Stampfl, Eudald Casals, Albert Duschl, Victor Puntes, Gertie J Oostingh

**Affiliations:** 1 Department of Molecular Biology, University of Salzburg, 5020 Salzburg, Austria; 2 Institut Català de Nanotecnologia (ICN), 08193 Bellaterra, Spain; 3 Institute of Toxicology, Helmholtz Centre Munich, 85764 Neuherberg, Germany

**Keywords:** Silver nanomaterials, wires, spheres, A549, lung cells, toxicity, immunomodulation, calcium imaging

## Abstract

**Background:**

In nanotoxicology, the exact role of particle shape, in relation to the composition, on the capacity to induce toxicity is largely unknown. We investigated the toxic and immunotoxic effects of silver wires (length: 1.5 - 25 μm; diameter 100 - 160 nm), spherical silver nanoparticles (30 nm) and silver microparticles (<45 μm) on alveolar epithelial cells (A549).

**Methods:**

Wires and nanoparticles were synthesized by wet-chemistry methods and extensively characterized. Cell viability and cytotoxicity were assessed and potential immunotoxic effects were investigated. To compare the effects on an activated and a resting immune system, cells were stimulated with rhTNF-α or left untreated. Changes in intracellular free calcium levels were determined using calcium imaging. Finally, ion release from the particles was assessed by ICP-MS and the effects of released ions on cell viability and cytotoxicity were tested.

**Results:**

No effects were observed for the spherical particles, whereas the silver wires significantly reduced cell viability and increased LDH release from A549 cells. Cytokine promoter induction and NF-κB activation decreased in a concentration dependent manner similar to the decrease seen in cell viability. In addition, a strong increase of intracellular calcium levels within minutes after addition of wires was observed. This toxicity was not due to free silver ions, since the samples with the highest ion release did not induce toxicity and ion release control experiments with cells treated with pre-incubated medium did not show any effects either.

**Conclusions:**

These data showed that silver wires strongly affect the alveolar epithelial cells, whereas spherical silver particles had no effect. This supports the hypothesis that shape is one of the important factors that determine particle toxicity.

## Background

Materials within the nanosized range in two dimensions are widely studied for their unique properties [1]. Nanowires are considered as potential building blocks for the next generation of optic, electronic, sensing, filtering and catalytic devices [2,3]. Silver is of interest because it has the highest electrical (6.3 ×10^7 ^S/m) and thermal conductivity (429 W/(m·K)) among all metals and it is well known for its biocidal activity [4,5]. Furthermore, it is at present the most used element in commercially available nanomaterial-containing products [6]. However, the increased application of silver wires is not sufficiently accompanied by the study of their potential impact on human health, whereas studies on spherical silver nanoparticles are profuse [7].

As the respiratory system is one of the major entrance sites for nanomaterials, several studies have focussed on research related to inhalation exposure or lung tissues. Several *in vivo *inhalation studies using rodents were performed; these studies showed that inhalation of silver nanoparticles (max. concentration 3 × 10^6 ^particles/ml) did not induce acute effects and only moderate effects after prolonged exposure times [8 - 12]. Inhalation of particles did result in accumulation of silver to secondary tissues such as the liver [8,9,11]. Moreover, oral administration of silver nanoparticles (14 ± 4 nm) to rats resulted in distribution of silver to the liver and kidneys, and was also found in lung, muscle and brain tissue [13]. *In vitro *studies have reported decreased cell viability, membrane leakage and increased ROS production after exposure of various cell lines, including lung cells, to silver nanoparticles [7,14,15]. The toxicity of silver nanoparticles was shown to be size-, rather than particle number-dependent, whereby smaller particles showed a higher toxicity when administered at the same mass concentrations [16,17]. This was attributed to the increased surface area and particle number and to the possibility that smaller particles evade macrophage clearance and readily diffuse into deeper tissues [18].

There are few toxicity studies using wire-shaped silver materials, but other wire-structured nano- and micro-objects are known to have fibrogenic and immunogenic effects [19-21]. The proposed mechanisms behind this toxicity are diverse [21], CNT and asbestos can induce frustrated phagocytosis in macrophages [22], but non-phagocytic cells are most likely affected in a different manner. For example, Casey at al. [23] and Guo et al. [24] showed that untreated CNTs can affect cell viability via medium depletion.

The role of fibre length is not established. Some studies describe enhanced responses with increasing lengths [20,22]. In contrast, other studies did not find such differences [25,26]. Furthermore, bio-persistence of the fibres seems to play an important role in their toxicity [20,27]. These studies indicate that there is a large difference in the response against wire-structured materials depending on the cell types, test models and wire types chosen.

According to findings on silver and fibre toxicity, silver wires might exert synergic toxic effects when introduced to the human body. *They theoretically combine the antimicrobial effects of silver with the toxicity of fibres*, resulting in nanomaterials that might be more harmful than their spherical counterparts. In addition, metallic silver may have a high durability and can remain in the body for a prolonged time. The investigation of potential human health risks caused by silver wires to evaluate their safety for future applications is therefore of importance.

In the presented work, the effects of polyvinylpyrrolidone (PVP)-coated silver wires on human alveolar epithelial cells (A549) were studied and the results were compared to those obtained with PVP-coated spherical silver particles and PVP-coated microparticles. Epithelial cells are the first to come into contact with inhaled particles in the proximal alveolar region, where the highest deposition of particles entering the lung takes place. Therefore, the activation of alveolar type II epithelial cells, as represented by the A549 cell line, has been hypothesised to serve as a direct and sensitive predictor of particle-induced inflammation [28].

The particle number was adjusted and the concentration curves widened to ensure that the different samples had an overlap concerning particle number, mass and surface area, whenever possible. Cell viability and cytotoxicity was tested as well as cytokine promoter (IL-6, IL-8 and TNF-α) and NF-κB binding sequence activation. Calcium imaging was used to investigate changes in intracellular free calcium levels ([Ca^2+^]_i_) of cells treated with silver wires, since increased levels of calcium are toxic for the cell and can result in both necrosis and apoptosis [29]. Finally, ion release by the silver materials in the suspension was tested using inductively coupled plasma mass spectrometry (ICP-MS) and the effects of the released ions on cell viability and LDH release were determined.

## Methods

### Nanomaterials: synthesis and characterisation

**Figure 1:**
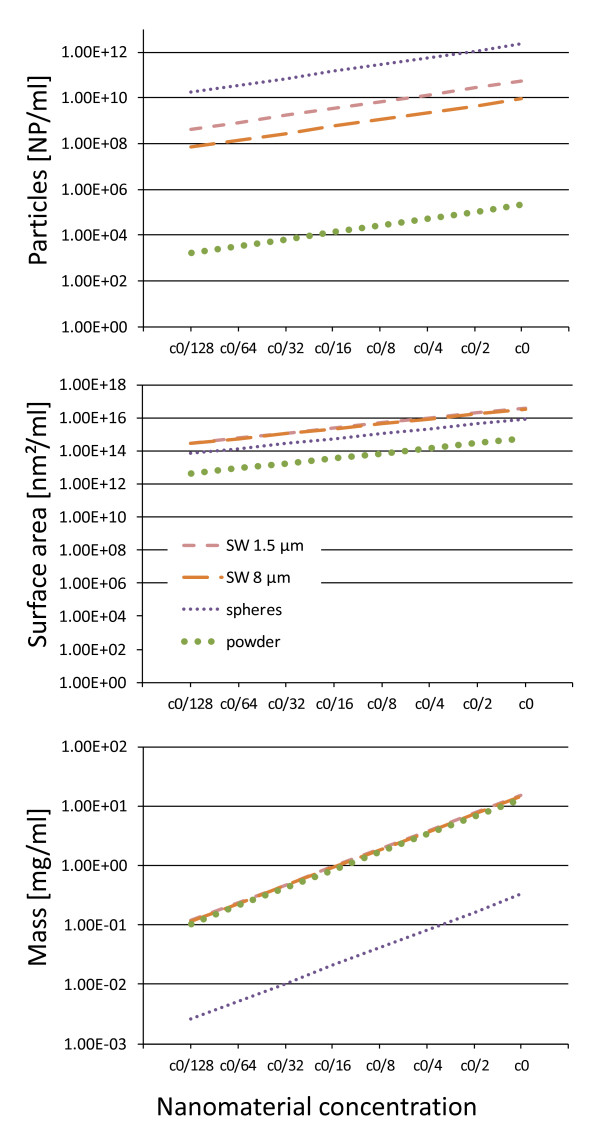
**Particle concentrations**. To illustrate the overlap in concentrations between the different nanomaterial preparations, the three graphs present particle number concentration vs dilution, surface area vs dilution and mass concentration vs dilution of selected silver wire types, spherical nanoparticles and microparticles.

Eight different silver wire preparations with sizes ranging from 1.5 - 25 μm in length and 100 - 160 nm in diameter, silver powder (<45 μm diameter) and spherical silver nanoparticles (30 nm diameter) were used. An overlap in mass concentrations as well as particle surface was used for exposure studies whenever possible (Figure 1 and Table 1).

**Table 1:** Sizes and highest concentrations (c0) of the different silver material preparations used.

Name	Length	Diameter	[particles/ml]	[nm²/ml]	[mg/ml]
**Spheres**	-	30 nm	2.3·10^12^	9·10^15^	0.33
**Powder**	-	< 45 μm	2.1·10^5^	5.9·10^14 +^	13.5
**SW 1.5 μm**	max. 1.5 μm	152 nm	5.5·10^10^	4.1·10^16^	15.47
**SW 5 μm**	max. 5 μm	126 nm	1.8·10^10^	3.6·10^16^	11.84
SW 6.5 μm*	6.5 μm	102 nm	9.1·10^9^	1.9·10^16^	5.055
**SW 8 μm**	max. 8 μm	158 nm	9.1·10^9^	3.6·10^16^	14.88
**SW 12a μm**	max. 12 μm	106 nm	6.8·10^9^	2.7·10^16^	7.52
**SW 12b μm**	max. 12 μm	140 nm	6.8·10^9^	3.6·10^16^	13.17
SW 12c μm*	12 μm	132 nm	9.1·10^9^	4.6·10^16^	15.26
**SW 25 μm**	max. 25 μm	115 nm	6.8·10^9^	5.5·10^16^	16.47

SW = silver wire. * used for calcium imaging only. ^+^Calculated surface area, for the microparticles the aggregation will have reduced this value.

Silver wires and spherical nanoparticles were synthesized by wet chemistry methods under conditions that ensured sterility and reproducibility. The reagents were purchased from Sigma- Aldrich (Vienna, Austria) and used as received. Glass material was sterilized and depyrogenated prior to use in order to reduce the levels of biological contaminants. All nanomaterials were coated with 0.2 M polyvinylpyrrolidone (PVP 55K) to make them biocompatible and to keep them dispersed in water. To remove free silver ions, the samples were washed three times and no instrument known to contain silver was used after this purification process.

Silver wires were synthesized using the polyol protocol [30]. Under magnetic stirring, 3 ml 0.1 M silver nitrate ethylene glycol solution and 3 ml 0.2 M PVP ethylene glycol solution were injected using a two-channel syringe pump into 5 ml of preheated ethylene glycol (160°C) at a rate of 300 μl/min. The reaction was continued for one hour at 160°C. To separate the ions from the wires, the samples were diluted five times by volume with acetone and were centrifuged several times at 2000 rpm for 20 min. Finally, the purified silver wires were redispersed in deionized water. Platinum traces were used to improve the control of the generated sizes of silver wires [31]. The length of silver wires was controlled by adjusting the ratio of PVP to silver nitrate and by using a specific reaction temperature.

The 30 nm silver nanoparticles were synthesized by the reduction of a silver salt with 0.01 M sodium citrate. PVP (0.2 M) was added to a washed sample of silver nanoparticles in aqueous media. The sample was dialyzed for 48 hours against pure water to remove free citrate from the solution. Under these conditions, the balance between free and absorbed citrate is lost and citrate is gradually replaced by PVP at the particle surface.

Silver microparticles (powder, <45 μm, ≥99.99% trace metals basis) were purchased from Sigma- Aldrich (#327107) and dispersed in distilled water at a concentration of 0.01 M. Thereafter, 0.2 M PVP was added, and the mixture was stirred for 4 hours. Finally, the mixture was washed three times. All silver materials were fully characterised as synthesised (10-fold more concentrated as the concentrations mentioned in Table 1) and in complete A549 cell culture medium (concentrations as mentioned in Table 1) by means of different techniques including transmission electron microscopy (TEM) using a JEOL 200 kV JEM-2011 (JEOL Ltd., Tokyo, Japan), zeta potential (surface charge) measurements and dynamic light scattering using a Malvern Zetasizer nano ZS (Malvern Instruments GmbH, Herrenberg, Germany) and UV-VIS spectrophotometry using a Shimadzu UV-2401 PC UV-VIS spectrophotometer (Shimadzu Europa GmbH, Duisburg, Germany) and these methods were employed as described earlier [32]. The obtained characteristics of the particles are stated in Table 1. The particles suspensions used before addition of the cell culture media were the highest concentrations that could be obtained without causing agglomeration of particles. The only exception was the commercially obtained silver powder; in this preparation a certain degree of sedimentation was always observed.

For each silver material type a dilution series of seven subsequent 1:1 dilutions in sterile water (ACILA^® ^LRW, LAL reagent water, ACILA AG, Mörfelden-Walldorf, Germany) was prepared (Figure 1).

### Cell lines

The human alveolar epithelial lung carcinoma cell line A549 (ATCC, LGC Standards GmbH, Wesel, Germany) was used. In addition, four different A549 reporter cell lines were used, these cell lines possess a luciferase reporter gene under the regulation of the promoter of a specific cytokine (interleukin (IL)-6, IL-8 or tumour necrosis factor-α (TNF-α)) or under regulation of four copies of the nuclear factor kappa B (NF-κB) response element. The establishment of these cell lines has been described in detail [33]. These cell lines have previously been used for the analysis of immunotoxic effects induced by metal(oxide) nanoparticles [34].

### Cell culture conditions and media

All cells were cultured at 37°C in a humidified incubator with 5% CO_2_. A549 cells were grown in RPMI 1640 without L-glutamine (PAA, Pasching, Austria), supplemented with 1% L-glutamine, 100 U/ml Penicillin, 100 μg/ml Streptomycin and 10% foetal calf serum (FCS, all from PAA). The transfected cell lines pIL-6 A549, pIL-8 A549 and pTNF-α A549 were cultured in A549 medium supplemented with 1% G-418 sulphate (50 mg/ml, PAA) as selection antibiotic. The NF-κB-luc transfected cells were purchased from Panomics (Affymetrix, Inc., Santa Clara, CA, USA) and cultured according to the distributor's description in DMEM without L-glutamine and sodium pyruvate (PAA), supplemented with 100 U/ml Penicillin, 100 μg/ml Streptomycin, 10% FCS (all PAA) and 0.2% Hygromycin B (50 mg/ml in PBS, GIBCO BRL^®^, Invitrogen GmbH, Lofer, Germany).

### Cell viability and cytotoxicity assays

A549 cells were trypsinised and diluted in A549 medium to a concentration of 2 × 10^4 ^cells/ml. A volume of 100 μl cells was plated into 96-well flat-bottom cell culture plates (Costar^®^, Corning Incorporated, Corning, NY, USA) and left over night in the incubator to allow them to adhere and regain their normal cell morphology.

The next day, the medium was changed to reset the levels of the analysed factors to zero. Half of the cells were treated with 20 ng/ml recombinant human TNF-α (rhTNF-α, ImmunoTools, Friesoythe, Germany) to simulate an ongoing immune response; the other half was left untreated. After a short recovery time of approximately 15 minutes, 10 μl of silver materials at concentrations as indicated for each experiment, or solvent or medium (as negative controls) were applied to each well (= 9% by volume). The cells were left to incubate at 37°C/5% CO_2 _for 0, 24 and 48 hours. All experiments were carried out for 6 independent wells.

#### Cell viability

Cell viability was assessed using the CellTiter-Blue^® ^cell viability assay kit from Promega (Madison, WI, USA). This assay is based on the ability of viable cells to convert the blue redox dye resazurin into the fluorescent compound resorufin whereas non-viable cells do not produce any fluorescent signal. A volume of 20 μl CellTiter-Blue^® ^Reagent was added to each well and the cells were left to incubate for 1 hour at 37°C. Fluorescence was recorded at 560_Ex_/590_Em _using a plate reader (Infinite^® ^200, Tecan, Grödig, Austria).

#### Cytotoxicity

Cytotoxicity was analysed by measuring the release of lactate dehydrogenase (LDH) using the CytoTox 96^® ^Non-Radioactive Cytotoxicity Assay Kit from Promega. This assay is based on the conversion of the tetrazolium salt 2-(4-Iodophenyl)-3-(4- nitrophenyl)-5-phenyl-2H-tetrazolium chloride (INT) into a red formazan product when LDH is released. A volume of 30 μl cell culture supernatant was transferred to transparent flat-bottom 96-well microtiter plates (Nunc MaxiSorp^®^, eBioscience, San Diego, CA, USA). Thereafter, 30 μl substrate were added and left to incubate in the dark for 30 min at room temperature. To stop the reaction, 30 μl stop solution were added. Absorbance was measured at 490 nm within 1 hour after adding the stop solution using a plate reader (Infinite^® ^200, Tecan).

### Cell morphology

To observe changes in cell morphology, cells were seeded in 24-well flat-bottom cell culture plates (Corning) at a density of 2 × 10^4 ^cells/ml one day prior to the addition of silver preparations. The next day, cells were treated with silver wires or microparticles for 0 to 48 hours and observed under an inverted optical microscope (Olympus IX70, Olympus Austria GmbH, Vienna, Austria).

### Immunotoxic effects

Immunotoxic effects were assessed using stably transfected A549 cell lines as previously described [33]. As a control for nanomaterial interference, the pIL-6 A549 cell line was used. About 10 minutes after addition of the nanomaterials, the samples were centrifuged at 1200 rpm for 5 min (25°C) in order to accelerate the settling of the silver wires, thereafter, the luciferase assay was performed. Experiments were carried out for 3 independent wells.

### Calcium imaging

Calcium imaging was used to investigate possible changes in intracellular calcium levels in A549 cells after treatment with silver wires. Preliminary studies showed that spherical silver nanoparticles did not induce any change in the calcium flux, therefore only two samples of PVP-coated silver wires with lengths of 6.5 and 12 μm (SW 6.5 μm and SW 12c μm) and PVP alone (control) were tested. The A549 cells were seeded on glass slides at least 24 hours prior to the experiment. Immediately before each experiment, cells were loaded with the Ca^2+^-sensitive fluorescent dye Fura-2- acetoxymethyl ester (Fura-2AM, Invitrogen GmbH) at a final concentration of 16.6 μg/ml for 20 minutes and washed in Krebs-Henseleit bicarbonate buffer (KHB, containing 1.2 mM Ca^2+^, 5.9 mM KCl, 1.2 mM MgSO_4_, 115 mM NaCl, 25 mM NaHCO_3_, 11.1 mM glucose and 1% BSA, adjusted to pH 7.4 by carbonation, purged with carbogen (95% O_2_, 5% CO_2_)) for 10 minutes. The glass slide was then inserted into the measuring chamber and suitable cells were selected for microscopic analysis. After positioning the injector, the fluorescence settings were adjusted. The exposure times for the excitation wavelengths (340 nm and 380 nm; exposure time ratio 1.5:1) were set by using the TILLVisION v4.01 software (TILL Photonics Imaging System Software, TILL Photonics GmbH, Gräfelfing, Germany). The illumination interval was set to 5 seconds and emission was measured at 510 nm. Additionally to fluorescence pictures, an infrared (IR) picture was taken at the same interval.

#### Physiological calcium conditions

To ensure that the cells had a steady baseline level of intracellular calcium, they were superfused with KHB containing 1.2 mM Ca^2+ ^for the first 3 - 5 minutes of each experiment. Subsequently, they were treated with 2 μM PVP in KHB (a concentration likely to occur if the coating agents detach from the silver materials) or the different silver wire samples, which were diluted in 1.2 mM Ca^2+ ^KHB to 1 × 10^10 ^Wrs/ml and added under static conditions using a syringe that was only connected to the injector pump when needed for injection. After exposure (~ 20 - 30 min), the cells were rinsed with 1.2 mM Ca^2+ ^KHB to wash out the effect. Calcium concentrations were assessed by measuring the fluorescence intensity continuously throughout the experiment. At least 3 independent experiments were performed in parallel by two different scientists.

#### Low calcium conditions

To investigate whether the changes of calcium concentrations were due to extracellular calcium influx or to depletion of internal calcium reservoirs, measurements were repeated with low calcium buffer. Cells were first exposed to 1.2 mM Ca^2+ ^for 3 minutes, and then to 0.1 mM Ca^2+ ^KHB until a steady baseline occurred. The silver wire samples were diluted to 1 × 10^10 ^Wrs/ml in KHB containing 0.1 mM Ca^2+ ^and added onto the cells as described above. Afterwards, the cells were first washed with 0.1 mM Ca^2+ ^KHB and then with 1.2 mM Ca^2+ ^KHB. Microscopic imaging was performed as described above.

### Ion release measurements

One day prior to the addition of the silver materials, a volume of 1 ml cells at a density of 5 × 10^4 ^cells/ml was plated into 24-well flat-bottom cell culture plates (Costar^®^, Corning Incorporated). The next day, cells were stimulated as described for cell viability and cytotoxicity assays and treated with 100 μl undiluted silver material preparations (= 9% vol.) for 48 hours. Following this exposure, the supernatants were carefully collected in 1.5 ml tubes. Supernatants were stored at -80°C until further analysis.

Ion release from the silver materials was assessed using inductively coupled plasma mass spectrometry (ICP-MS). For this, silver objects in the supernatant samples were separated from free ions by means of centrifugation. The particle pellet was discarded and analysis of the silver ion content in complete cell culture media was performed using an Agilent 7500cx ICP-MS (Agilent Technologies, Santa Clara, CA, United States) with a detection limit of 2.2 × 10^-10 ^M (= 23.86 ng/l).

### Ion release control experiments

In order to determine whether ions or PVP released from the particles had any effect on cell viability or cytotoxicity, wires, microparticles or nanoparticles as well as appropriate controls (medium, solvent, PVP) were diluted in cell culture medium at the highest concentration used in the experiments and incubated at 37°C/5% CO_2 _for 48 hours. After this incubation time, the particles were removed from the supernatant by high speed centrifugation and the supernatants were added to A549 cells (after removing all the cell culture medium from the cells) that were plated out the previous day as described above. The 8 μm long wires (SW 8 μm) were included as positive control. After an additional 48 hours of incubation, cell viability, LDH release and total LDH (cell number) were analysed. Experiments were carried out for 3 independent wells.

### Statistical analysis

For cell viability, cytotoxicity and immunotoxicity experiments, each condition was measured for 3 to 6 independent wells. Results were expressed as the mean value ± standard deviation (stdev) calculated using Microsoft^® ^Office Excel. *P*-values were obtained by employing the Student's *t*-test and *p*-values <0.05 were regarded as statistically significant.

For calcium imaging, analysis was performed using TILLVisION v4.01 software. After marking a suitable area for background subtraction, the cells were selected manually and tagged for analysis. By taking the ratio (ratio 340/380), the program eliminated interfering factors like bleaching or uneven dye distribution. To create graphs, the data was then imported to Microsoft^® ^Office Excel.

## Results

The aim of this study was to investigate the influence of shape on particle toxicity. Human alveolar epithelial cells (A549) were exposed to wire-shaped or spherical PVP-coated silver micro- and nanoparticles, and their effects on cell viability, cytotoxicity and immunomodulation were analysed.

### Particle characterisation

**Figure 2:**
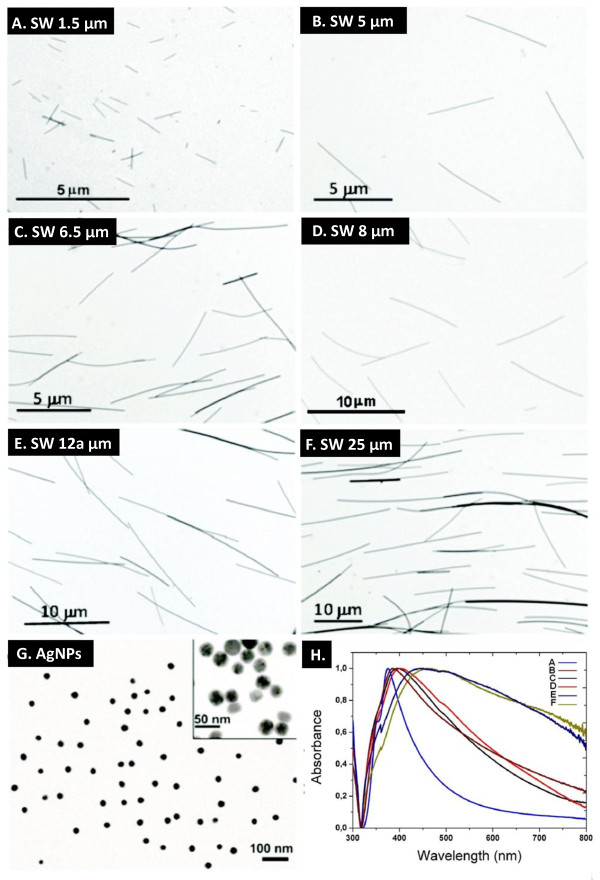
**Particle characterisation**. A-G: Transmission electron microscopy was used to characterize wire length and particle diameter. Images of SW 1.5 μm, SW 5.0 μm, SW 6.5μm, SW 8.0 μm, SW 12a μm and SW 25 μm and AgNPs of 30 nm diameter are shown. H: The optical absorption spectra show the resonance peaks (transverse dipole resonance) of the silver wires of Figure 2A-F by which their diameters were determined.

Silver particles and wires were characterised by TEM, UV-visible spectrophotometry, surface charge measurements (zeta potential) and DLS. From images obtained by TEM (Figure 2A-G) the average diameters and lengths of the silver materials were determined. The diameters were then confirmed using UV-visible spectrophotometry. In Figure 2H the optical absorption spectra for the silver wires depicted in 2A-F are shown. For all silver wire preparations the zeta potential was measured directly after synthesis and after diluting in cell culture medium (9% v/v). The obtained values were similar for all wires, as synthesized a mean zeta potential of -31 mV was found and this value turned to -13.5 mV in the presence of complete cell culture medium. All silver wires and nanoparticles were found to be mostly monodispersed and showed little agglomeration. The only exception was the silver powder for which a certain degree of sedimentation was always observed (not shown).

### Assay interference and consistency of cell number

**Figure 3:**
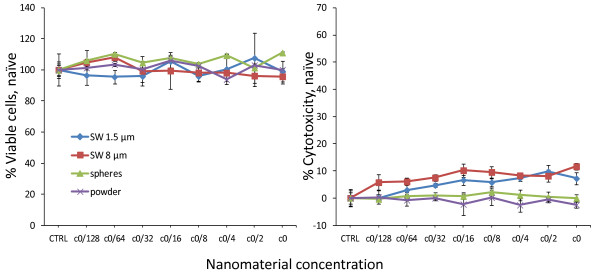
**Analysis of assay interference and cell number consistency**. Interference of the different silver materials with the CellTiter-Blue^® ^(left panel) and LDH release (right panel) assays was tested by measuring 15 minutes after addition of the materials. Data are presented as mean ± stdev of 3 independent wells. Furthermore, these data show that the cell number was constant at the start of the experiment.

All materials were tested for eventual assay interference by analysing the different read-out parameters after 0 hours of incubation. No interference was detected for any of the particles tested (Figure 3). These data also showed that the cell number was the same in all samples at the beginning of the analysis. A decreased cell number would result in a lower metabolism of the dye per well and thus in a decreased fluorescence measurement when using the CellTiter-Blue^® ^test.

### Cell viability

**Figure 4:**
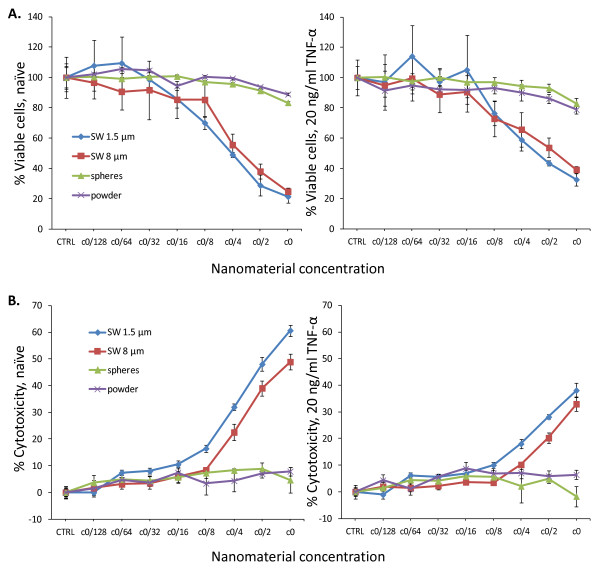
**Effects of the silver materials on A549 cell viability and their cytotoxic potential**. A. Cell viability of naïve cells (left panel) and cells stimulated with 20 ng/ml rh-TNF-α (right panel) was assessed after 48 h incubation using the CellTiter-Blue^® ^assay from Promega. Data are presented as mean ± stdev of at least 6 independent wells. B. Cytotoxicity of the different silver preparations was tested after a 48 h incubation using the CytoTox 96^® ^Non-Radioactive Cytotoxicity Assay Kit from Promega. Data are presented as mean ± stdev of 6 independent wells.

The effect of the silver preparations on the viability of the cells was determined using the CellTiter- Blue^® ^assay. Addition of silver wire preparations to A549 cells significantly decreased the cell viability in a concentration dependent manner by maximally ^~^80% (Figure 4A). This effect was observed after 24 hours and became more pronounced after 48 hours. Cells treated with 20 ng/ml rhTNF-α were less affected compared to naïve cells and the viability was decreased by maximally 60-70% as a result of treatment with the silver wires. However, the difference between naïve and rhTNF-α treated cells was only significant for SW 1.5 μm (p < 0.05), but not for SW 8 μm. For spherical nano- or microparticles a slight decrease could be observed, and the cell viability remained at about 80-90% for both naïve and rhTNF-α treated cells.

### Cytotoxicity

To analyse the cytotoxicity induced by the different silver preparations, the LDH release assay was used (Figure 4B). After measuring LDH release from cells treated with silver nanomaterials, the LDH release was converted to cytotoxicty by normalizing all LDH release values to the control cells, which were set to 0% cytotoxicity. At high silver wire concentrations (c0/4 and higher, 2.25 × 10^9 ^- 1.35 × 10^10 ^Wrs/ml, 9 × 10^15 ^- 1.01 × 10^16 ^nm²/ml, 3.68 - 3.83 mg/ml), enhanced amounts of LDH were released by naïve cells and the effect was most pronounced for the smaller wires (60% cytotoxicity for SW 1.5 μm, 50% for SW 8 μm; p < 0.001). RhTNF-α stimulated cells incubated with silver wires released less LDH compared to naïve cells, and the difference between naïve and stimulated cells was significant (p < 0.01 for SW 1.5 μm, p < 0.001 for SW 8 μm). However, the difference in toxicities between the 1.5 and 8 μm sized wires was not as pronounced as that observed in the naïve cells (p < 0.005). A very slight but significant increase in cytotoxicity could be observed for the microparticles (p < 0.001 for naïve cells and p < 0.005 for rhTNF-α treated cells), whereas spherical nanoparticles did not significantly affect the cells. In addition, significant differences could be observed between rhTNF-α stimulated cells treated with micro- and nanoparticles (p < 0.05), but not for naïve cells. The results obtained with the LDH-release assay largely confirmed the results obtained with the CellTiterBlue^® ^assay.

### Morphological changes

**Figure 5:**
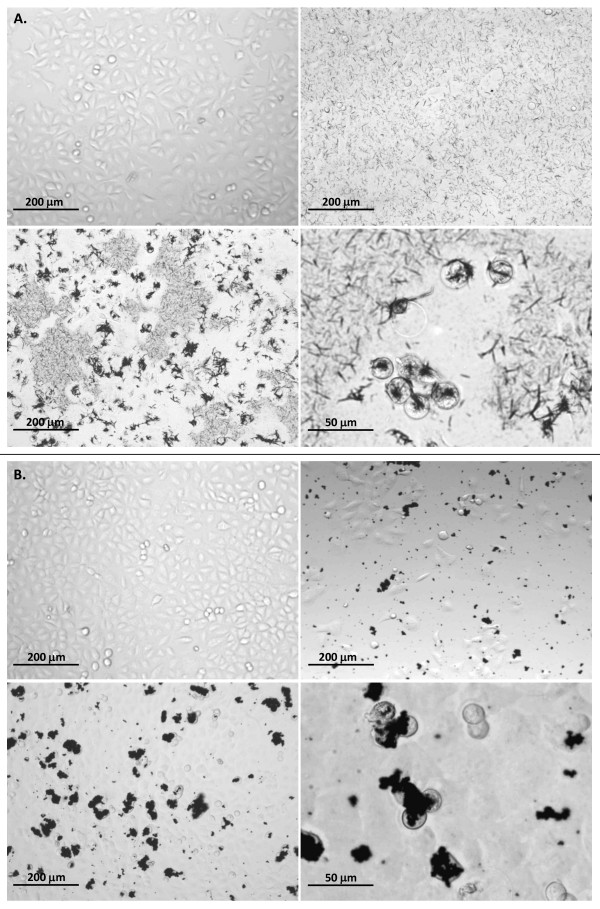
**Interaction of silver wires and microparticles with A549 cells**. A. Microscopic images were taken at 100× magnification after 24 h for untreated cells (control, upper left panel), and 15 min (upper right panel) and 24 h (lower left panel) after addition of 1.5 μm silver wires (SW 1.5 μm, final concentration 2.3 × 10^10 ^Wrs/ml, 1.7 × 10^16 ^nm²/ml, 6.46 mg/ml). After 24 h, enhanced agglomeration of wires on cells could be observed and the cells rounded up (lower left panel, 400×). B. Microscopic images were taken at 100× magnification after 24 h for untreated cells (control, upper left panel), and 15 min (upper right panel) and 48 h (lower left panel) after addition of microparticles (final concentration 3 × 10^4 ^particles/ml, 8.3 × 1013 nm²/ml, 1.9 mg/ml). Rounded up cells and agglomeration of particles on cells could also be observed after 48 h (lower right panel, 400×).

Morphological changes of cells exposed to silver wires and microparticles were observed using an inverted optical microscope. After 24 hours incubation with silver wires, the A549 cells rounded up and detached from the plastic support. An enhanced agglomeration of the wires on the cells could be observed, suggesting an interaction between the cells and wires. The results shown as images taken at different time points are depicted in Figure 5A for SW 1.5 μm. All other wires had similar effects (data not shown).

For the microparticles, a certain degree of agglomeration could always be observed, even directly after addition to the culture. After 48 hours incubation the sedimentation/agglomeration was even more prominent. Furthermore, some of the cells rounded up after 48 hours compared to the control (Figure 5B). Interestingly, there were often large numbers of particles attached to the cells with a round morphology, it is likely that these cells form the dead population measured above.

### Immunotoxic effects

Immunotoxic effects were analysed using stably transfected A549 reporter cell lines. NF-κB activity decreased at high silver wire concentrations, and rhTNF-α treated cells were slightly less affected than naïve cells (p < 0.01 for SW 1.5 μm and p < 0.0001 for SW 8 μm, data not shown). Furthermore, a small but significant difference was observed for rhTNF-α stimulated cells treated with 1.5 μm and 8 μm silver wires, where the smaller wires had a larger impact (p < 0.05).

For spherical and micro-sized particles no statistically significant effects were found. Overall, the reductions largely followed the cytotoxic effects and are most likely due to cell death. In addition, the regulation of several cytokine promoters was tested (pIL-8, pIL-6, pTNF-α) and these were all affected in a similar manner as the NF-κB binding sequence induction (data not shown).

### Calcium imaging

**Figure 6:**
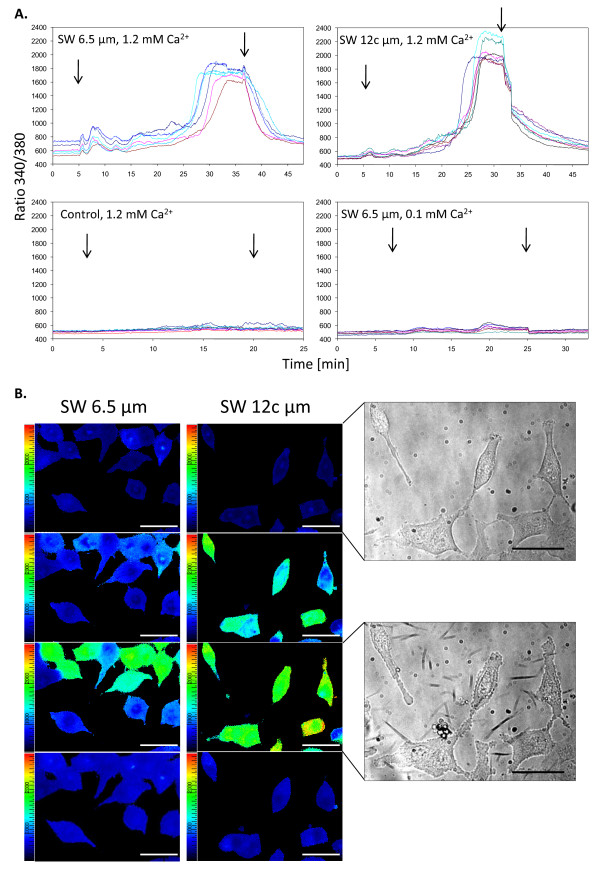
**Calcium imaging of silver wire-treated A549 cells**. A. Cells were treated with 6.5 μm (SW 6.5 μm, upper left panel) and 12 μm (SW 12c μm, upper right panel) long silver wires at a concentration of 1 × 10^10 ^Wrs/ml in 1.2 mM Ca^2+ ^buffer and fluorescence intensities were measured at 340 and 380 nm excitation. PVP was used as control (lower left panel) and additional experiments were carried out in low calcium buffer (lower right panel) to investigate whether changes were from extracellular calcium influx or intracellular storage depletion. Arrows indicate the addition and elution of particles/coating agent. Each line represents the response of one single cell. B. False- colour fluorescence images were generated by taking the 340/380 nm ratio pixel by pixel after background subtraction. Images were taken 5, 26, 32 and 47 minutes after the start of the experiment using 6.5 μm wires (SW 6.5 μm) at a concentration of 1 × 10^10 ^Wrs/ml (left panel) and 4, 26, 30 and 47 minutes after the start of the experiment using 12 μm wires (SW 12c μm) at the same concentration (middle panel). Blue = low calcium levels, red = high calcium levels. Infrared pictures (right panel) were taken immediately before and 25 min after addition of 12 μm (SW 12c μm) long wires. Scale bars represent 50 μm.

Changes of intracellular free calcium ([Ca^2+^]_i_) levels in A549 cells were detected by calcium imaging using the calcium-sensitive fluorescent dye Fura-2AM. A two-fold increase of [Ca^2+^]_i _levels was induced by 6.5 μm long silver wires (SW 6.5 μm) after 20 - 25 minutes of incubation in 1.2 mM Ca^2+ ^buffer (Figure 6A, upper left panel) and could be removed by washing with KHB. Even stronger effects were observed for 12 μm long wires (SW 12c μm), whereas treatment with PVP alone did not induce such an effect. Furthermore, treatment of cells with silver wires in low [Ca^2+^] buffer (0.1 mM Ca^2+^) did not cause any significant increase of intracellular calcium levels, as shown for SW 6.5 μm (lower right panel), indicating that the extracellular calcium influx is the cause of the increased [Ca^2+^]_i _levels.

The changes of [Ca^2+^]_i _levels in cells treated with silver wires in 1.2 mM Ca^2+ ^buffer were also illustrated by false-colour fluorescence images (Figure 6B). Here, the increase was indicated by the change in colour from dark blue (low calcium) to light green/red (high calcium). After washing the cells with KHB, the calcium content returned to values close to its base level, confirming the transient nature of this increase. The increase was slightly higher for cells treated with 12 μm long wires compared to 6.5 μm long wires.

In the infra-red pictures (Figure 6B, right panel) morphological changes and signs for apoptotic stress, such as membrane blebbing, could be observed. Additionally, cell swelling could be seen for some of the cells (not shown).

### Ion-release data

The release of silver ions from the different silver materials was assessed by measuring the concentration of silver ions in the cell culture media after 48 hours of incubation using ICP-MS (Table 2). For most of the silver preparations the values were <10 mg/l. The only sample in which a higher concentration of silver ions was detected (25.2 mg/l) was the one in which Ag powder was added to medium without rhTNF-α.

**Table 2:** Silver ion concentration in cell culture media after 48 hours incubation at 37°C/5% CO_2_.

Sample	mg/l Ag^+^
SW 5 μm + rhTNF-α 48 h A549	3.89
SW 5 μm 48 h A549	2.67
SW 8 μm + rhTNF-α 48 h A549	0.80
SW 8 μm 48 h A549	0.69
SW 12b μm + rhTNF-α 48 h A549	2.96
SW 12b μm 48 h A549	4.82
Ag Powder + rhTNF-α 48 h A549	7.42
Ag Powder 48 h A549	**25.2**
30 nm AgNPs (citrate coated) in cCCM	3.6

### Ion release control experiment

**Figure 7:**
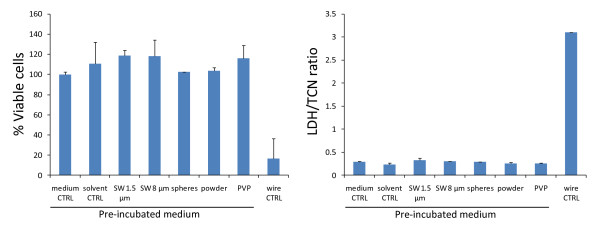
**Ion release control experiment**. Particles were incubated in cell culture medium for 48 hours at 37°C/5% CO_2 _and then removed by centrifugation. The supernatants were added onto A549 cells, incubated for another 48 hours and analysed for cell viability and cytotoxic effects using CellTiter Blue^® ^(left panel) and CytoTox 96^® ^Non-Radioactive Cytotoxicity Assay Kit from Promega (right panel), respectively. Data are presented as mean ± stdev of 3 independent wells.

In order to determine whether ions or PVP released from the particles had any effect on cell viability or cytotoxicity, particles were incubated in medium for 48 hours and the supernatants were then added onto cells for another 48 hours. The results of this ion release control experiment showed that the supernatants never induced toxicity or LDH release whereas the 8 μm long wires (SW 8 μm, included as positive control) did induce cytotoxicity and cell death (Figure 7).

## Discussion

In this study the influence of shape and size of silver particles on human lung epithelial cells was investigated, because of the high interest in nanowire containing products [1] and because published data on fibre toxicity [20] have shown that wire-shaped objects can be more harmful than spherical ones when administered at the same dose. Additionally, silver is often assumed to be toxic and bioactive.

Our results showed that the PVP-coated spherical silver particles had no impact on the cells, whereas wire-shaped objects did induce a strong toxicity at similar particle mass, surface areas and number concentrations. We did not find any cytotoxicity or loss of cell viability when testing medium preconditioned with silver preparations, showing that the observed effects were not due to released silver ions. Foldbjerg et al. found that A549 cells exposed to Ag^+ ^showed toxic effects on mitochondrial function at doses of 1-10 μg/ml, with a LD_50 _around 6 μg/ml [14]. Even though the concentrations determined in the cell culture supernatants were in average 3 times higher (see Table 2), the kind of exposure completely differs when comparing direct spiking with a slow release of ions from nanoparticles. It is likely that the cells can cope with a small amount of silver ions, whereas an immediate addition of a high concentration (as occurs when adding AgNO_3_) of the same ions can affect the cells.

Our data are in contrast to other studies, where toxic effects of spherical silver nanoparticles on alveolar epithelial cells were observed [14,16]. The discrepancy between these studies can be explained by a difference in the silver ion concentrations, but these values were not always tested. In addition, PVP coating might have made the silver materials more biocompatible. However, in a previous study we used citrate coated silver nanospheres and these did also not induce any cytotoxicity or decrease in cell viability (data not shown). Furthermore, PVP was used as coating agent by another group using THP-1 cells and their data showed that these particles were toxic [35]. In addition, lack of colloidal stability could also be the reason for silver toxicity.

The cytotoxic effects found here are likely due to a direct interaction between the wires and the cells, since no specific immunotoxic responses could be observed. The imaging data clearly showed a direct contact and accumulation of wires onto the cells, this interaction might cause toxicity by inducing small punctures in the cell membrane. In contrast, small spherical particles can be engulfed (most likely via endocytosis) [36,37], and the cell membrane closes again, whereas microparticles are not taken up at all. Interestingly, cells stimulated with TNF-α showed less reduced cell viability and less cytotoxicity. Since it is known that stimulation with TNF-α can trigger the activation of NF-κB, which then mediates the transcription of genes associated with proliferation, immune responses and cell survival [38], TNF-α-induced NF-κB activation might in part explain the enhanced survival of TNF-α treated cells.

Concerning the impact of length, the acquired data are ambiguous. With respect to cytotoxicity and immunotoxicity, there is a tendency that the shortest wires affected the cells to a greater extent than the longer wires. However, the data obtained from calcium imaging showed the opposite effect. Therefore, wire length may affect early and late phase responses of alveolar epithelial cells differently, but this aspect has to be studied in more detail.

Elevated calcium levels can be associated with oxidative stress and cell death [29]. The transient increase of intracellular calcium after treatment with silver wires was often accompanied by cell swelling and morphological changes such as membrane blebbing, showing that cellular stress occurs rapidly after contact between silver wires and cells. Consistent with another study using particles under low calcium conditions [39], we conclude that extracellular calcium influx is the cause of the observed calcium levels.

Translating these data to potential health effects that might occur *in vivo *is still a challenging task and much research has to be dedicated to finding the appropriate *in vitro *models that can predict events taking place *in vivo*. Especially since not all novel nanomaterials can be tested *in vivo*, for logistic, ethical and financial reasons.

## Conclusion

Our data showed that spherical silver nano- and microparticles had almost no impact on A549 cells, whereas wire-shaped silver objects induced a strong cytotoxicity, loss in cell viability, and early calcium influx. These data indicate that shape is indeed one of the important factors that can determine toxicity. In contrast, the wire length did not have a major effect on the level of toxicity. We did not find any immunotoxic effects of the silver wires or particles, except for those that were directly linked to decreased cell viability. However, the addition of rhTNF-α seemed to have a protective effect on cells.

The increased toxicity and the absence of specific immunotoxic responses could imply that the toxicity of the silver wires is caused mechanically by the needle-like structure. The employed cell line (A549) does not have phagocytic activity, but A549 cells were shown to take up particles, most likely via endocytosis [36,37]. We propose that the wires, that have a very small diameter of around 100 nm, might try to enter the cells. The large length of the wires, when compared to the nanoparticles, does not allow complete entry and this induces cell membrane damage. Moreover, the physical presence of the wires impairs repair of the damage and this results in cell death.

Addition of rhTNF-α might in part protect the cells by stimulating repair mechanisms within the cells (mediated by NF-κB). The above hypothesis will be the basis for further studies.

Finally, risk assessment of silver wires is still in its early stages of development, but strongly necessary as illustrated by the presented data, and this study is a starting point to initiate further research regarding the possible toxic effects of silver wires for human health.

## Competing interests

The authors declare that they have no competing interests.

## Authors' contributions

LCS performed the cell viability, cytotoxicity and immunotoxicity experiments and was involved in the calcium flux experiments. She was also heavily involved in the preparation and revision of the manuscript. EG prepared the silver preparations, performed the particle characterization and ICP-MS measurements, and wrote the parts about the particle synthesis and characterisation. AS was involved in the experimental design and exertion of the calcium flux experiments and critically revised the manuscript. EC participated in the synthesis of the silver preparations and the particle characterisation and was involved in the revisions of the manuscript. AD was involved in the planning of the study, obtained funding for the project and critically revised the manuscript. VP was involved in the experimental design of the particle synthesis and characterisation and critically revised the manuscript. GJO was involved in the experimental design of the cell viability, cytotoxicity and transfected cell line based assays and the coordination of the study and was heavily involved in the preparation and revision of the manuscript. All authors read and approved the final manuscript.
